# The formation mechanism of “sports fandom circle” in the digital media era: An analysis from the perspective of fan emotional dynamic development

**DOI:** 10.1371/journal.pone.0330900

**Published:** 2025-09-03

**Authors:** Yuan Cheng, Yin Wu

**Affiliations:** School of Economics and Management, Shanghai University of Sport, Shanghai, China; Çanakkale Onsekiz Mart University: Canakkale Onsekiz Mart Universitesi, TÜRKIYE

## Abstract

The rise of the “sports fandom circle” has become a significant phenomenon in the digital media era, reshaping the emotional and social dynamics of sports fan communities. This study employs grounded theory methodology to analyze web-scraped data from 40 selected accounts on major Chinese social media platforms [Weibo, Xiaohongshu, and Bilibili] over a two-year period. These accounts were identified based on their focus on celebrity athletes [e.g., Fan Zhendong, Sun Yingsha, Wang Chuqin] and their engagement in fan-specific activities such as support, fan clubs, and voting. The analysis involved open coding, axial coding, and selective coding to construct an emotional interaction model. Our findings reveal that international sports events serve as emotional catalysts, where competition, nationalism, and athlete narratives trigger fans’ emotional arousal. Digital media platforms, including spatiotemporal extension, virtualization, and selectivity, amplify these emotions, fostering proximal emotional attachment to athletes. Through symbolic interactions and digital consumption, fans transition from individual engagement to collective emotional aggregation, solidifying their sense of group identity. However, in the reproduction process, the anonymity of digital media platforms can lead to emotional polarization, intensifying conflicts among fan groups, or facilitate emotional resocialization, fostering more rational and inclusive fandom behaviors. This study provides a theoretical framework for understanding the emotional evolution of sports fandom in the digital media era and offers practical insights for managing online fan communities, mitigating conflicts, and promoting a healthier digital sports culture.

## 1. Introduction

The women’s singles table tennis final at the 2024 Paris Olympics, contested between Chinese players Chen Meng and Sun Yingsha, garnered significant public attention [1]. What should have been a high-level yet enjoyable spectator event was marred by disruptive audience behavior. During the match, the overwhelming support from the live audience went to Sun Yingsha, while cheers for Chen Meng were noticeably rare. After Chen Meng’s victory, a flood of aggressive comments emerged online targeting her and her coach—some even containing extreme expressions like “go die.” More alarmingly, some fans stalked the athletes, severely violating their privacy. These manifestations of toxic behavior within the “sports fandom circle” have prompted public concern.

The concept of “sports fandom circle” refers to fan communities characterized by intense idolization and emotional investment in sports celebrities [2]. These groups operate in ways strikingly similar to entertainment fandom, engaging in characteristic behaviors such as organized support campaigns, online opinion control, and popularity ranking activities [3]. Against the backdrop of proliferating digital media platforms that increasingly blur the boundaries between physical and virtual worlds [4], these fan communities have rapidly emerged as a novel trend in digital socialization. Compared to traditional sports fans, the “sports fandom circle” demonstrate a higher degree of organization and broader societal influence [3]. While this new fan structure has amplified public engagement with athletic events and athletes, their increasingly extreme and irrational behavior has exceeded reasonable boundaries. In response, on August 17, 2024, the Chinese Table Tennis Association issued a statement, sternly pointing out that the “fandom circle” chaos disrupts training and competition order, endangers the work and lives of athletes and coaches, and creates a negative social impact, vowing to hold those responsible accountable according to the law. The statement not only directly responded to the extreme fan behavior during the Olympics, but also highlighted that the “sports fandom circle” is deviating from the healthy spirit of sports and urgently requires attention.

A pertinent question arises: how does a traditional fan subculture evolve into the “sports fandom circle”, a form distinguished by its powerful organizational mobilization and profound public influence? Previous studies have predominantly examined sports fan behavior through the frameworks of social interaction theory and collective action theory. The former emphasizes that the behavior of sports fan communities is generated through interaction: fans stimulate emotional resonance through symbolic practices such as cheering and dressing up at live events, strengthen their sense of belonging through online discussions and collaborations, and construct their identity through continuous communication, thereby laying the foundation for organized behavior [5–7]. The latter emphasizes that sports fan groups engage in coordinated actions around common goals, such as supporting teams, protesting commercialized football, or expressing political stances, and through these collective practices, fans strengthen group identity and cohesion, further enhancing organizational mobilization and social influence [8–11]. Although existing studies provide important perspectives on the organizational logic, emotional responses, and identity construction of sports fans, it predominantly focuses on the overt behaviors at the group level, treating emotions as a result of behavior, and overlooks the key role of individual emotions as a driving force in the construction of group organizations.

Moreover, as a product within the context of sports culture, the emotional mechanism of “sports fandom circle” is distinctive. As Skinner and Stewart [12] point out, sports itself has a highly ritualized competition structure, strong national identity symbols, and a clear competitive logic of wins and losses, all of which provide fans with a stronger space for emotional investment and make it easier to connect emotions with social identity, public values, and even political stances. In contrast, “entertainment fandom circle” mainly rely on stars and works to sustain emotions, and their influence on public issues and connection to national identity are relatively weaker [13], which makes the “sports fandom circle” not simply a replica of the “entertainment fandom circle”. Therefore, studying the unique emotional formation and development of fans in “sports fandom circle” is of great significance for understanding their group culture and mobilization capacity.

The rise of “sports fandom circle” emerged in the new realm of the digital media era. Social media platforms have broken through the limitations of traditional media’s one-way communication, providing a multidimensional, immediate, and interactive space for emotional expression [14]. Fans are no longer passive spectators but actively shape the narrative of events through diverse emotional expressions [15]. This platform-based empowerment mechanism may reshape fans’ emotional practices and modes of communication [16]. Thus, when studying the emotional development of fans in the “sports fandom circle”, it is essential to fully consider the new arena of the digital media era. In summary, this study aims to examine the formation and reproduction mechanisms of the “sports fandom circle” by focusing on the dynamic development of fan emotions within the context of digital media. The goal is to deepen the understanding of fan behavior logic, expand the theoretical scope of sociocultural research in sports, and offer new perspectives for relevant governance practices. Based on this framework, the study focuses on two key research questions:

[1] What are the key factors influencing the emotional development of fans within the “sports fandom circle” in the digital era?
[2] What is the dynamic path of emotional development of fans within the “sports fandom circle” in the digital era?

## 2. Literature review

### 2.1. Conceptualizing of “sports fandom circle”

The “fandom circle” originates from popular culture, centered around interest-based communities that revolve around idols through production and consumption, symbolic texts, ritualized interactions [such as comment control, fan support, and ranking activities], and internal organizational mechanisms [13]. The “sports fandom circle” is an extension of entertainment fandom into the field of sports, forming a subcultural sphere driven by fan cohesion. Its defining characteristics include idolization and worship of sports stars, intense and sustained emotional investment, and irrational behaviors [17]. Specifically, “sports fandom circle” is centered on emotional bonds, with fans developing emotional connections with athletes or teams out of admiration, not only focusing on match results but also identifying with their idols’ charisma and personal stories [18]. Secondly, “sports fandom circle” rely on collective behavior, as fans interact through social media, forums, and other platforms to form virtual communities that transcend geographical and cultural boundaries, with their engagement further reinforced through offline activities and merchandise purchases [19]. Lastly, “sports fandom circle” exhibit a strong sense of production, with fans expressing their admiration through secondary creations such as short videos and images, contributing to the dissemination of sports culture [20].

The earliest concept established within sports fan culture is the “sports enthusiasts”, which revolves around sporting events and emphasizes competition and rational analysis. In contrast, “sports fandom circle” resemble celebrity fandom, focusing more on individual sports stars [3]. Research shows that social media platforms amplify the personal influence of these athletes [21]. On platforms like Weibo’s sports super topics, fans engage in content creation and interaction around specific athletes, forming an individual-centered fan culture that is further reinforced through organized support campaigns, comment management, and voting [22]. When a sports fan’s emotional investment in an athlete surpasses rational engagement, they may transition into “fandom circle”. This study, through the observation of fan communities surrounding table tennis athletes Fan Zhendong, Sun Yingsha, and Wang Chuqin, identifies two primary groups within the “sports fandom circle”: those who purely idolize sports stars and those who simultaneously identify as fans and sports enthusiasts. Therefore, when analyzing the “sports fandom circle”, it is essential to consider its distinctive emotional identification and support mechanisms, rather than equating it with general sports enthusiasts.

### 2.2. “Sports fandom circle” in the digital media era

Current research on sports fans primarily focuses on social identity, collective action, fan loyalty, fanatic behavior and fan communities. Social Identity Theory suggests that sports fans develop a sense of group belonging through their identification with a particular team or athlete, establishing boundaries between the “in-group” and the “out-group”, with this identity reflected not only in their viewing behavior but also extending to daily social interactions, further strengthening their emotional connection to their supported team or athlete [23,24]. Based on this, Collective Action Theory emphasizes that sports fans are not only individuals expressing emotions but also groups with organizational and mobilization capabilities as they enhance their influence, secure resources, or impact decision-making through collective actions such as cheering and social media campaigns while the development of social platforms has made these actions more networked and collaborative, influencing public discourse and the commercial market [8,25]. The studies have further explored the formation mechanisms of fan loyalty. Based on an study of Turkish football fans, Kural and Özbek [26] found that fans’ sense of social identity is a key antecedent to team loyalty, which in turn directly influences their consumption intentions—highlighting the transformation of emotional attachment into consumption behavior. Similarly, Yun et al. [27] using Australian fans as their sample, expanded the understanding of loyalty drivers by identifying team brand image, fan engagement, satisfaction, and enduring involvement as significant factors influencing fan loyalty. With the deepening commercialization and globalization of sports, fanatical behavior has become a research focus [28]. Due to their strong emotional investment in athletes, fans actively participate in both online and offline support activities, purchase merchandise, and follow competitions on tour. Such behaviors not only strengthen the connection between fans and their idols, but also promote the development of sports consumption, event marketing, and the sports-related cultural tourism economy [26,29]. Moreover, existing studies has begun to focus on the structure and mechanisms of interaction within sports fan communities. Asada and Ko [30] pointed out that the relative size of the community and its sense of organization serve as important foundations for sports socialization, as these structural characteristics influence members’ sense of identity and belonging. Mastromartino and Zhang [31] further emphasized that such identity and belonging can stimulate fans’ emotional satisfaction and group affiliation, thereby enhancing their loyalty to sports brands and willingness to consume, ultimately promoting sustained engagement and behavioral transformation. Meanwhile, Yoshida et al. [32] revealed that both formal and informal factors—such as brand equity and community rituals—jointly drive fan community identification, which in turn influences fans’ engagement, sense of responsibility, and communicative behaviors. In the digital environment, Kirkwood et al. [33], through an analysis of online sports communities, identified multiple fan role types within virtual communities, constructed a role classification framework, and revealed the various functions and influences that different types of fans assume in these communities. However, despite existing studies analyzing the behavioral characteristics of sports fans from multiple dimensions, certain limitations remain. These studies primarily focus on traditional sports fans centered around sporting events, highlighting competitive elements [34], team affiliation [35], and consumer behavior [26], while paying limited attention to the “sports fandom circle”—fans centered on individual athletes, marked by strong emotional dependence and idolization. Nevertheless, these studies have laid an important foundation for research on the “sports fandom circle”, particularly by establishing theoretical frameworks in areas such as group identity, emotional investment, social interaction, and organizational behavior, thus providing academic support for further understanding its formation mechanisms.

“Sports fandom circle” has attracted widespread public attention in China. Some scholars have begun to explore its formation through factors such as capital involvement [13,36], and socio-cultural transformation [19,37]. Capital intervention has significantly contributed to the commercialization of sports idols by enhancing their visibility through commercial packaging and market operations, with sponsorships, endorsements, and event promotions further increasing their exposure, while fans engage emotionally by consuming tickets, merchandise, and other related products, thereby strengthening their emotional bonds and promoting the development of the “sports fandom circle” [13,19,36]. Additionally, the growth of the “sports fandom circle” has been accelerated by socio-cultural changes, with young people in the digital age seeking identity and emotional fulfillment through sports idols, and as idol culture spreads, fans place greater emphasis on emotional connections with their idols, transforming the fandom into a platform for cultural identity [37,38]. Although previous studies have explored the formation of “sports fandom circle” from various perspectives, most are based on static viewpoints, and systematic research on the dynamic development of fan emotions in the digital media era remains relatively lacking. For instance, how fan emotions evolve over time, the depth of emotional resonance, and the interaction mechanisms between emotion and behavior have not been sufficiently investigated, particularly in the context of the digital media era.

With the development of digital media, users are immersed in an increasingly real-time and diverse online space, fostering new models of fan emotion development, where the scope, intensity, and forms of emotional expression have undergone significant changes [39]. Research indicates that social media and live streaming platforms provide users with instant channels for emotional expression and sharing, enhancing emotional resonance and community cohesion [40]. The widespread adoption of user-generated content [UGC] enables fans to strengthen their community identity in a self-organized manner, while algorithmic recommendations and big data make emotional experiences quantifiable—for example, by generating engagement scores based on likes, shares, and comments to identify “core fans” and “potential users,” enabling targeted advertising and merchandise promotion that transforms emotional value into commercial value [41]. As a result, digital technology not only reshapes how fan emotions are expressed but also profoundly influences their formation and evolution. Against this backdrop, fan emotions exhibit a dynamic nature. However, while existing research has laid a theoretical foundation for understanding the formation of sports fan communities, it primarily adopts a static perspective on fan behavior, lacking sufficient consideration of the dynamic evolution of fan emotions in the digital media environment. The dynamic nature of this emotional development calls for more in-depth research to uncover the underlying psychological and social mechanisms, thereby enriching the understanding of the “sports fandom circle” as a cultural phenomenon.

However, traditional fan culture theories face dual limitations in explaining contemporary phenomena within the “sports fandom circle”: first, limited theoretical adaptability to changes in the media environment; second, insufficient attention to the Chinese socio-cultural context. From the perspective of media evolution, take Jenkins’ theory of “textual poaching” as an example—it emphasizes fans’ active interpretation and re-creation of media texts, wherein fans assign new meanings to original works through practices like fan fiction [42], yet fails to account for the passivity and tendency toward homogenized interpretations shaped by algorithmic content delivery on digital platforms. Similarly, Fiske’s theory of “fan productivity,” which focuses on non-commercial cultural creations driven by fans’ personal interests, highlighting their resistance to dominant cultural meanings through spontaneous community practices [43], does not adequately capture production behaviors in the “sports fandom circle” that are deeply influenced by capital logics—such as data-driven support campaigns and consumption of commercial endorsements. Moreover, constrained by specific historical conditions, fans under the subcultural theoretical perspective have typically been regarded as fragmented communities situated at the margins of social life [44], with insufficient recognition of their capacity for collective action and organization. Thus, fandom theories rooted in traditional media struggle to account for platform-dominated and commercially driven practices in contemporary “sports fandom circle”.

Moreover, the “sports fandom circle” in China exhibits distinct local characteristics, particularly in emotional expression, organizational mobilization, and fan practices. For example, emotional expression among Western sports fans tends to emphasize individual emotional release [45], whereas in the Chinese context, it stresses emotional uniformity, with sentiments often disciplined as part of organized support efforts [46]. In terms of mobilization, Western sports fans activities are typically driven by personal interest and spontaneous community engagement, lacking the highly organized structures [36,47]. The “sports fandom circle” in China tends to operate in a “project-based” manner, relying on fan leaders or support teams to devise plans and coordinate execution [37,38]. At the level of fan practice, Western sports fans are more inclined to discuss athletic performance and less involved in organized online opinion management [48]. In contrast, the “sports fandom circle” in China has developed highly systematized digital behaviors such as “data ranking” and “comment control” [20,37,46]. These differences highlight the collective, structured, and digitalized nature of the “sports fandom circle” in China. However, most existing theories are rooted in Western contexts, and their analytical frameworks struggle to fully capture the complexity and particularity of the “sports fandom circle” in China.

In conclusion, existing theories face dual limitations—of media logic and cultural context—when explaining the formation path and emotional structure of the “sports fandom circle”. On this basis, it is necessary for this study to break away from pre-established theoretical structures and adopt a grounded theory approach, starting from real fan practices within the Chinese social media context to deeply explore the internal mechanisms underlying the formation of the “sports fandom circle”. This not only helps to address the shortcomings of existing theories regarding new media and indigenous cultural dimensions, but also offers potential for theoretical innovation in understanding affective communities in the context of digital China.

## 3. Materials and methods

### 3.1. Research method

This study adopts the Grounded Theory approach to explore the key influencing factors and generative pathways of emotional development among fans in the sports fandom subculture. The choice of Grounded Theory is based on the following three considerations:

First, this study focuses on the processual issue of how fan emotions develop within sports fandoms, with particular emphasis on identifying the key influencing factors and mapping their developmental trajectories. However, academic research on sports fandom is still in its early stages, especially in terms of fan emotional construction, where there is a lack of systematic theoretical support and mature analytical frameworks. Existing literature largely focuses on surface-level descriptions of fan behaviors [26,49,50], and the pathways through which fan emotions develop in interaction have not been systematically explored. Therefore, this study cannot directly apply existing theoretical models, but rather needs to “ground” new concepts and theoretical frameworks in empirical materials.

Second, as a qualitative research method, Grounded Theory emphasizes the abstraction of concepts from first-hand data and the gradual construction of theory through systematic comparison, categorization, and theoretical abstraction [51]. Its core advantage lies in its independence from pre-established theoretical frameworks, instead identifying the causal mechanisms and dynamic processes underlying complex social phenomena through a three-stage coding procedure—open coding, axial coding, and selective coding [52]. This makes it particularly suitable for topics where mechanisms are unclear and theory has yet to be established [53], which aligns with the exploratory nature, undefined variables, and unknown pathways of this study.

Finally, in terms of existing research practice, Grounded Theory has been widely applied in fields such as education [54], management [55], and online education development [56], and has also been extensively adopted by scholars in the field of sport studies to explore issues such as motivation for sport participation [57], identity construction and sports fan subculture formation [58], Sports skill acquisition [59], and individual development pathways [60,61]. This demonstrates its effectiveness in revealing complex social behavioral processes. With the aid of Grounded Theory, this study can systematically analyze the emotional construction pathways of sports fandom fans across different platforms, interactive contexts, and community structures, providing theoretical innovation for understanding the formation patterns of sports fandom.

In conclusion, given the complexity of the research subject, the limitations of existing theories, and the pathway-oriented nature of the research question, Grounded Theory provides an appropriate analytical paradigm and theoretical construction approach for this study.

In the development of grounded theory, three major methodological schools have emerged. The classical grounded theory, represented by Glaser, emphasizes the natural emergence of theory from data [62]; Strauss advocates a procedural approach guided by pragmatism [63]; and Charmaz develops constructivist grounded theory, highlighting participants’ perspectives and the co-construction of meaning [64]. After a comparative assessment, this study adopts the procedural grounded theory approach represented by Strauss, based on the following considerations:

First, procedural grounded theory supports the researcher’s role as a “critical participant.” It enables the systematic analysis of complex and unstructured social media texts from the “sports fandom circle” through structured coding procedures—open coding, axial coding, and selective coding—to uncover the logic behind emotional development. At the same time, it acknowledges the researcher’s involvement and reflexivity in theory construction, encouraging vigilance toward personal biases or preconceptions about fandom, thereby ensuring the academic validity of the resulting theory. Second, this approach emphasizes the refinement of dynamic processes and causal logic, aligning closely with the present study’s focus on the formation mechanisms of the “sports fandom circle”. By mapping out developmental trajectories and constructing causal pathways, it becomes possible to effectively trace the evolution of fan emotions within social contexts—making this approach particularly well-suited to mechanism-focused research compared to other schools of grounded theory. Third, the method stresses that theory generation should emerge from real-world contexts and allows researchers to enter the research process with problem awareness, fostering dialogue with existing theories. This pragmatic orientation not only aligns with the theoretical aims of this study but also supports the further development of theory on the foundation of fan culture research. Finally, procedural grounded theory has already been successfully applied by scholars in related fields such as fan culture [65,66] and online communities [67,68], demonstrating its theoretical relevance and practical effectiveness in addressing complex sociocultural phenomena. This further validates the methodological soundness and feasibility of the research design adopted in this study.

### 3.2. Data collection

This study employs the Python programming language in combination with the Selenium and Beautiful Soup libraries to implement a web crawler for collecting data related to the “sports fandom circle” on Chinese social media platforms. The crawler script is designed with keyword-based search rules [e.g., “Fan Zhendong,” “Wang Chuqin,” “Sun Yingsha”] to extract fan-generated content—including posts, comments, reposts, and interactions—within a specified time frame. Metadata such as post timestamps and user engagement metrics are also retained to ensure both the breadth and analyzability of the dataset.

#### 3.2.1. Platform selection.

This study utilizes web scraping techniques to collect data, focusing primarily on three major Chinese social media platforms: Weibo, Xiaohongshu, and Bilibili. These platforms were selected for the following reasons. First, the “sports fandom circle” demonstrates high activity levels, diverse interaction formats, and strong fan clustering effects on these platforms, providing rich and varied data sources. Second, compared to other platforms such as Douyin, Zhihu, or Tieba, these three offer greater advantages in terms of fan organization, sustained engagement, and the depth of textual content, making them more suitable for capturing fan emotional expression and behavioral practices. Third, the user demographic on these platforms is predominantly between the ages of 18 and 40, which closely aligns with the core participant group of the “sports fandom circle”, thereby ensuring representativeness. The characteristics of the three platforms are as follows:

Weibo, as one of the largest social media platforms in China, integrates text, image, and video sharing with commenting and reposting features. It is highly real-time and viral, making it a key venue for sports event discussions and fan emotional expression. Its “Super Topics” function creates a typical fan community space, where users engage in daily ranking, support activities, and emotional expression, thereby forming stable online communities. Weibo has become a crucial platform for fandom mobilization and public opinion dissemination; Xiaohongshu primarily features image-text posts and short videos, emphasizing “authentic sharing” and emotional expression. It has become an important space for sports fans to document viewing experiences, publish support content, and express identity, with particularly high usage among female fans; Bilibili is characterized by fan-created videos [e.g., compilations, voiceovers, commentaries] and real-time “danmu” [bullet screen] interactions. It serves as a typical platform for creative fan production and the construction of collective memory within the fandom.

#### 3.2.2. Fans selection.

This study focuses on fans within the “sports fandom circle” who support three specific athletes: Fan Zhendong, Sun Yingsha, and Wang Chuqin. These athletes’ fan groups were selected as research subjects because they possess large and active fan bases in China. Their outstanding performances in major domestic and international competitions, participation in social initiatives, and carefully crafted public images have sparked widespread discussion and attention. Fan Zhendong, Sun Yingsha, and Wang Chuqin are among the top players in Chinese table tennis. Their professional achievements, personal narratives shared on social media, engagement in public welfare, and frequent interactions with fans have drawn significant emotional investment from their supporters. Studying fan accounts related to these athletes helps us better understand the emotional development and group dynamics of the “sports fandom circle” in the digital media era.

#### 3.2.3. Accounts selection.

This study selected comments from 40 accounts within the “sports fandom circle” as the sample. The sample size was determined based on extensive preliminary searches and account screening, while fully considering the research objectives and the grounded theory’s requirements for case diversity and data saturation. The selection of accounts was based on purposeful sampling, which involves deliberately choosing cases that are representative and information-rich in relation to the research question [69].Based on the following criteria: clear idol focus—all selected accounts center on the three top athletes; prominent fandom characteristics—most accounts reflect fandom culture traits such as “support,” “voting,” and “guarding”; high activity level—selected accounts post frequently with rich content, with some Weibo accounts exceeding tens of thousands of posts; considerable fan base—follower counts range from tens of thousands to over a million, covering top-tier [e.g., official or core fans] and mid-tier accounts to reflect discourse and participation across different fan levels. Based on these criteria, 40 accounts with over 10,000 followers were ultimately selected. To further enhance the diversity of sample types, we supplemented fan accounts using keyword-based searches. Specifically, we employed advanced search functions on social media platforms to conduct combined queries, using keywords that included fan self-identifiers [e.g., “Sha fans,” “Fan stars,” “Big Head”] and fandom-specific terms [e.g., “guard” or “support”]. Based on fan tags, profile descriptions, and posting behaviors, we then assessed the accounts’ fan identities and characteristics, with particular attention to relatively marginal fan accounts that represented varying emotional stances and modes of participation—such as highly supportive, critical, or passively observant. In the end, 10 additional accounts were selected to serve as a meaningful supplement to the original sample. A full list of the accounts is provided in S1 Table.

#### 3.2.4. Ethical statement.

This study collected only publicly accessible user content from platforms such as Weibo, Xiaohongshu, and Bilibili, without involving any private information or restricted data. The data collection process adhered to the basic requirements of each platform’s terms of service and developer policies, with controlled retrieval frequency to avoid disruption to platform operations. All data were anonymized during analysis and used solely for academic research, in full compliance with ethical standards and research integrity guidelines.

### 3.3. Data analysis

**Fig 1:**
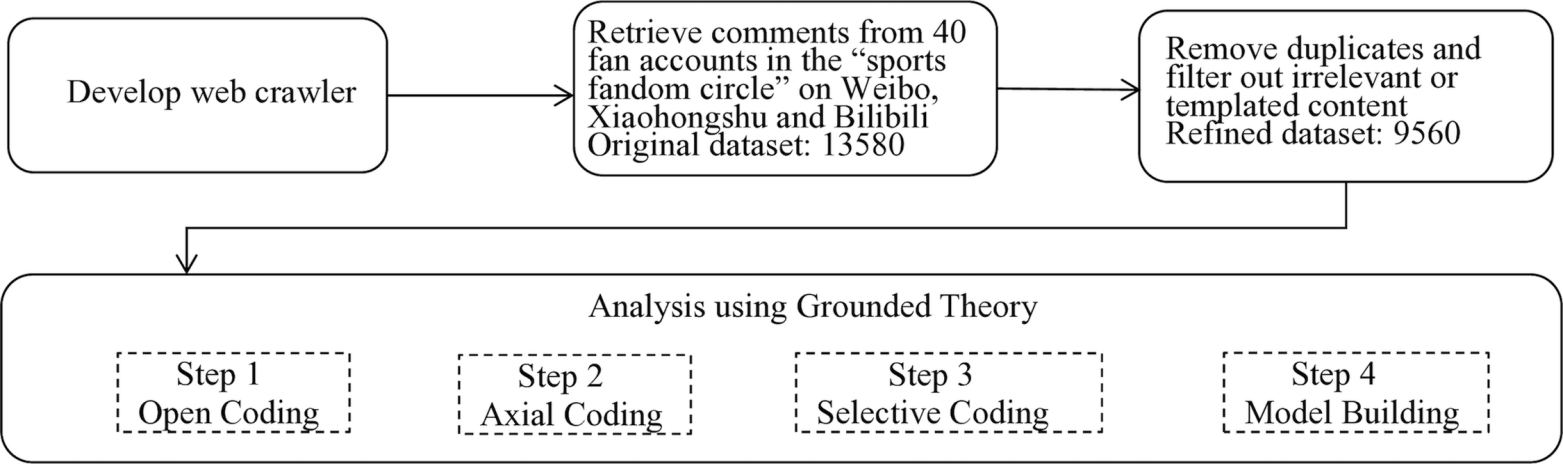
Flowchart of data collection, processing, and analysis.

Data collection focused on all content and comment interactions posted by these accounts over the past two years, yielding 13,580 raw comment entries. To improve data quality, we conducted a screening and cleaning process on the collected comments. Irrelevant content—such as advertisements and giveaway information—was removed, along with duplicate comments, templated phrases, and other invalid entries. Based on this, we further filtered the data using the criterion of “contextually meaningful.” Comments meeting any of the following characteristics were deemed valid for analysis: [1] focused on the core topic, such as interactions related to specific athletes or the “sports fandom circle”; [2] demonstrated emotional engagement, reflecting fans’ emotional tendencies [e.g., affection, support] and their expressive behaviors; [3] revealed “fandom circle” characteristics, such as organizational patterns, identity expressions, or discourse norms; [4] maintained contextual coherence, forming a semantically connected unit with surrounding comments to ensure completeness of meaning. After this process, a total of 9,560 valid comments were retained as the primary corpus for analysis in this study. All data were imported into NVivo 11 software for systematic coding and analysis. NVivo 11 is a widely used qualitative data analysis tool that is particularly well-suited for processing large-scale textual data [70]. The use of NVivo 11 enables efficient organization, categorization, and analysis of large volumes of text, supports complex coding procedures, and ensures the effective implementation of the grounded theory methodology. Through NVivo 11 analysis, researchers were able to more accurately identify emotional interaction patterns within fan communities and uncover potential organizational structures, thus providing strong empirical support for this study. The analysis followed a structured coding process consisting of the following steps: [1] Open coding: This involved a detailed examination of fan discussions related to specific athletes in order to identify initial concepts and categories. [2] Axial coding: Based on the open coding stage, connections between categories were established. Researchers identified six key processes that drive the formation and reproduction of the “sports fandom circle,” with emotional arousal, emotional expression, and emotional convergence forming the core process chain. [3] Selective coding: Finally, the categories were integrated to form a coherent theoretical model that explains how fans’ emotional experiences evolve and aggregate to form a shared community. The flowchart of data collection, processing, and analysis is shown in Fig 1.

#### 3.3.1. Open coding.

Open coding is the process of systematically analyzing raw data segment by segment to extract and label key concepts and themes, facilitating their categorization and conceptualization [71]. The process begins by identifying initial concepts from the raw data. For example, a statement such as “Pan Zhanle has made thorough preparations for the Olympics, with the goal of winning the gold medal” is abstracted into the initial concept of goal-oriented behavior. These initial concepts are then classified, and related concepts within the same category are grouped and assigned a representative name. Once the data content reaches a sufficient level of saturation, it is organized to ensure that it accurately reflects the mechanisms underlying the formation and reproduction of the “sports fandom circle”. The resulting dataset is designated as the “Example of the formation and reproduction mechanism for ‘sports fandom circle’” [see Table 1].

**Table 1:** Example of the formation and reproduction mechanism for “sports fandom circle”.

Data Coding	Data Records	Initial Concept	Initial Category
Code 1	When observing Fan Zhendong’s table tennis matches, he consistently demonstrates a strong competitive mindset, maintaining intense focus on his own performance while closely monitoring his opponent’s movements. Throughout the match, he exhibits unwavering commitment and effort, ensuring he remains fully engaged at all times.	competitive awareness	Competitive Attributes
Code 2	Every match featuring Sun Yingsha fills me with intense anticipation. Despite her exceptional skills, the Olympic stage is fraught with uncertainties, where even the slightest changes can lead to unexpected outcomes. This unpredictability evokes a mix of exhilaration and apprehension, adding to the emotional intensity of the experience.	Uncertainty	
Code 3	Each time I watch Pan Zhanle’s swimming competitions, I find myself eagerly hoping for him to achieve remarkable results and secure the gold medal. The anticipation is truly exhilarating and adds to the excitement of the experience.	Victory Expectation	
Code 4	In the lead-up to major competitions, I often feel particularly anxious, worrying about whether our athletes might make critical errors at decisive moments.	Competition Anxiety	
Code 5	Fan Zhendong’s matches against top-tier opponents are a captivating clash of strategy and power. Each of his returns is calculated to dominate his opponent, and the intense back-and-forth during the competition creates a tension so gripping that one cannot look away.	Competitiveness	
Code 6	At the Paris Olympics, competition results were almost simultaneously presented during the events. This real-time feedback allowed us to immediately experience the intensity and suspense of the matches as they unfolded.	Real-Time Feedback	
Code 7	The competitive atmosphere at the Paris Olympics was electrifying, with each match resembling an intense battle. The spectators’ cheers combined with the athletes’ focused expressions created a tension-filled yet passionate environment.	Competitive Atmosphere	
Code 8	Pan Zhanle has thoroughly prepared for the Olympics, with his sights firmly set on the gold medal. His focus and goal-oriented mindset during the competition are truly admirable, as if the medal he envisions is already within reach.	Goal-Oriented	

After organizing and deconstructing the raw data, the analysis adhered to a systematic procedure: “defining materials–identifying categories–naming categories–determining category properties–open coding–coding memos” [71]. Sentence-by-sentence coding was performed using Nvivo software. To ensure coding quality and reduce subjective bias, this study conducted three rounds of repeated comparison and cross-validation of the textual data. In each round, the researchers independently read the original materials and labeled initial concepts. After each round, the concepts were compared and revised. Ultimately, a total of 100 initial concepts were extracted and labeled using the format “b+number”, such as b1–b3: competitive awareness, victory expectation, competition anxiety and b9–b11: national emblem, national flag, national anthem. These initial concepts were further analyzed and consolidated into 37 subcategories, labeled in the format “B+number”. Subsequently, these 37 subcategories were compared and subjected to clustering analysis, resulting in the formation of 15 distinct initial categories [see Table 2].

**Table 2:** Open coding analysis of “sports fandom circle” formation and reproduction.

Initial Category	Subcategory	Initial Concept
A1 Competitive Attributes	B1 Internal Factors	b1-b4 Competitive Awareness, Victory Expectation, Competition Anxiety, Goal-Oriented
	B2 External Factors	b5-b8 Competitive Atmosphere, Oppositionality, Real-Time Feedback, Uncertainty
A2 Nationalist Symbols	B3 National Symbolism	b9-b12 National Emblem, National Flag, National Anthem, National Team Uniform
	B4 Linguistic and Cultural Symbolism	b13-b15 Native Language, Slogans and Mottos, Traditional Cultural Elements
	B5 Athlete National Identity Symbolism	b16-b17 Representing the Country in Competitions, National Sports Heroes
	B6 Political Nationalism Symbolism	b18-b20 International Rivalries, Symbolic Meaning of Hosting Rights, Historical Rivalries and National Sentiments
A3 Athlete Narratives	B7 Personal Experience	b21-b23 Growth Story, Adversity Experience, Struggle Journey
	B8 Sporting Spirit	b24-b26 Fair Competition, Teamwork, Perseverance and Effort
	B9 Role Model Influence	b27-b29 Public Image, “Hero” Role Model Effect, Social Responsibility
A4 Emotional Closeness	B10 Sense of closeness	b30 “Athlete as a Friend”
	B11 Sense of Resonance	b31-b33 Resonance with Athlete’s Struggle Story, Shared National Honor, Collective Passion and Dreams
	B12 Sense of Participation	b34 Involvement in Athlete’s Growth
A5 Emotional Attachment	B13 Trait Attachment	b35-b36 Recognition of Athlete’s Charismatic Personality, Admiration for Athlete’s Moral Qualities and Spirit
	B14 Achievement Attachment	b37 Admiration for Athlete’s Outstanding Athletic Achievements
A6 Symbolic Interaction	B15 Visual Symbols	b38-b40 Digital Emojis, Fan-Created GIFs, Event Posters on Social Media
	B16 Linguistic Symbols	b41-b43 Online and Offline Slogans and Chants, Fan Discussions on Social Media, Fan-Created Comments or Interactive Phrases
	B17 Behavioral Symbols	b44-b45 Pre-Competition Traditions, Online Interactive Activities
A7 Digital Consumption	B18 Content Consumption	b46-b48 Watching on Streaming Platforms, Subscribing to Event Content, Subscribing to Athlete Membership Content
	B19 Product Consumption	b49-b51 NFTs, Athlete Merchandise, Tickets [for Matches and Events]
	B20 Participation Consumption	b52-b54 Fan Support Activities, Ranking Support, Crowdfunding Projects for Athletes
A8 Fans Aggregation	B21Online Aggregation	b55-b57 Fan groups on Social Media, Online Live Interaction, Forum Discussions
	B22 Offline Aggregation	b58-b60 Collective Cheers at Live Events, Fan Meetups, On-Site Support at Offline Activities
A9 Group Identity Recognition	B23 Cultural Identity	b61-b63 Recognition of Fan Community Beliefs, Subcultural Identity [Sense of Belonging in Fan Activities], Cross-Cultural Identity [Recognition of Diverse Cultures within the Fan Community]
	B24 Social Identity	b64-b66 Emotional Support Among Fans, Shared Interests Among Fans, Sense of Unity Among Fans
	B25 Value Identity	b67-b69 Resonance with Idol’s Values, Pride in Supporting Idol’s Public Welfare Activities, Collective Pride in Idol’s Achievements
A10 Emotional Polarization	B26 Emotional Opposition Among Fan Groups	b70-b71 Formation of Opposing Factions Due to Different Stances, Intensified Emotional Hostility from Internal Factional Disputes
	B27 Emotional Conflict Between Fans and Athletes	b72 Fans’ Over-Idealization of Athletes and the Discrepancy Between Idealized and Actual Performance
	B28 Tension Between Fans and Commercial Brands	b73-74 Athlete Brand Endorsements, Event Brand Sponsorships
A11 Positive Reproduction	B29 Promoting Sports Fairness	b75-b77 Gender Equality in Sports Participation, Regional Equality in Sports Participation, Equal Voice in Sports Discourse
	B30 Disseminating Sports Culture	b78-b80 Promoting Positive Athlete Images and Stories, Spreading Sports Spirit and Values, Enhancing Public Attention and Participation in Sports Events
A12 Spatiotemporal Extensibility	B31 Temporal Extension	b81-b83 Ongoing Monitoring of Athlete Updates, Event Replays, Real-Time Discussions of Historical Events
	B32 Spatial Extension	b84-b85 Global Interactions in Online Communities, Cross-Regional Participation in Virtual Events
A13 Virtualization	B33 Content Virtualization	b86-b88 Digital Videos and Images of Events, Virtual Athlete Cards, Digital Athlete Stories
	B34 Communication Virtualization	b89-b90 Online Communication on Social Media Platforms, Event Updates Shared on Digital Platforms
A14 Selectivity	B35 Active Selection	b91-b93 Fans Actively Following Athlete Updates of Interest, Participating in Discussions of Specific Events, Choosing to Share Content that Aligns with Their Own Views
	B36 Algorithmic Recommendation	b94-b96 Content Pushed Based on User Interests, Content Suggested Based on User’s Past Behavior, Guided Content Recommendations
A15 Anonymity	B37 Identity Anonymity	b97-b100 Participation in Community Activities with Virtual Identities, Anonymous Posting of Event Insights, Anonymous Participation in Online Q&A, Anonymous Sharing of Event Memories

#### 3.3.2. Axial coding.

To establish connections between different categories and develop core categories, this stage adopts the axial coding method of grounded theory. The 15 initial categories will be repeatedly compared and analyzed to identify their underlying logical relationships. Ultimately, six main categories will be constructed: characteristics of international sports events, emotional arousal, emotional expression, emotional aggregation, emotional community reproduction, and features of the digital media platforms [see Table 3].

**Table 3:** Spindle coding analysis of “sports fandom circle” formation and reproduction.

Main Category	Initial Category	Category Connotation	Data Records
International Sports Events Characteristics	A1Competitive Attributes	The competitiveness in international sports events is embodied by athletes striving for victory in fair competition, driven by both internal and external factors.	*“During the men’s singles quarterfinal in table tennis, when Fan Zhendong was up against the Japanese player Tomokazu Harimoto, I didn’t dare blink for even a second—I just kept thinking, he has to win! It felt like I was right there fighting alongside him, completely forgetting that I was only watching a match.”*
	A2Nationalism Symbolism	International sports events serve as a significant platform for nationalism, represented through national symbols, culture, and athletes’ identities.	*“He wasn’t fighting for himself—he was fighting for the whole country. In my heart, he’s a national hero, a source of pride for our generation. In that moment, I genuinely wanted to rush onto the podium and give him a hug.”*
	A3Athlete Narratives	Event narratives revolve around athletes’ personal experiences and stories of struggle, evoking emotional resonance among the audience.	*“I saw a report saying that his family was not well-off when he was a child, and he would start training before dawn every day. That kind of relentless effort, rising step by step, really makes my heart ache—it helps me understand even more why he fights so hard.”*
Emotional Awakening	A4Emotional Closeness	Through the establishment of familiarity, resonance, and sense of participation, a closer emotional connection is formed between fans and athletes.	*“I can read one of his Weibo posts over and over again—it feels like he’s talking directly to me. More and more, he doesn’t seem like some distant superstar, but like someone from among us.”*
	A5Emotional Attachment	Fans’ attachment to athletes is manifested in their admiration and recognition of the athlete’s personality or achievements, leading to emotional dependence and sustained attention.	*“No matter whether he wins or loses, just seeing him smile on the court makes everything feel worthwhile. His resilience and gentleness have deeply moved me. Whenever I face difficulties in life and think of him, I feel like I’ve regained my strength. It’s as if he’s already become a part of my life.”*
Emotional Expression	A6Symbolic Interaction	Fans express emotions through visual, verbal, and behavioral symbols, enhancing interaction and participation.	*“Every time Fan Zhendong wins a match, I take a screenshot and turn it into a GIF—capturing that exact moment when he swings his paddle. When I post it, everyone says it’s so powerful. It feels like it conveys the excitement even better than just using words.”*
	A7Digital Consumption	Fans express their emotions toward athletes through digital consumption behaviors, such as watching live streams or purchasing virtual products.	*“I bought his limited edition NFT badge and the official 3D autograph card—even though I don’t really have a use for them, just having them in my collection makes me happy.”*
Emotional Aggregation	A8Fans Aggregation	The collective phenomenon of sports fans forming communities at events, activities, or digital platforms, manifested through interaction and emotional support.	*“The first time I watched Wang Chuqin’s match, I was surrounded by people wearing support jerseys, and we were all cheering together—it was absolutely electrifying.”*
	A9 Group Identity Recognition	Fans recognize themselves as part of a specific athlete’s or team’s fan group by identifying with certain culture, social relationships, and values, strengthening their connection and sense of belonging to the group.	*“No matter whether we’re from the south or the north, we’re all guardians of Fan Zhendong. We can’t bring negative attention to him, and everyone in the group consciously follows the rules.”*
Reproduction of Emotional Community	A10 Positive Reproduction	Emphasizes promoting sports fairness, disseminating sports culture, and increasing societal attention and participation in sports.	*“Stop fixating only on popular sports and athletes from powerhouse nations—athletes from impoverished regions are just as talented. We should strive to give them more chances to be seen.”*
	A11 Emotional Polarization	Fans’ emotional responses may become polarized, manifested through opposing emotional stances of support and opposition.	*“I truly believed he could carry the national table tennis team—I followed every match, and whenever others criticized him, I posted replies to defend him. But after getting knocked out in the Olympic round of 32 and losing several matches afterward, I was completely disappointed.”*
Digital Media Platforms Features	A12 Temporal-Spatial Extension	Digital media technology allows fans to transcend traditional time and space limitations, enabling them to engage in watching and discussing sports events anytime and anywhere.	*“Sometimes when I rewatch her matches from a few years ago and read the live comments [danmu], it feels like I actually experienced that victory together with her.”*
	A13 Virtualization	Digital media technology transforms physical entities into virtual content, enhancing the efficiency of communication and dissemination.	*“You can find match videos and photo galleries online with just a quick search—it’s really convenient to watch.”*
	A14 Selectivity	Users choose content or communities based on their interests, and algorithms recommend information that aligns with their preferences.	*“After I clicked on one of Fan Zhendong’s match videos, my homepage was flooded with his highlights and interviews for days. The platform really gets me—but honestly, I’m enjoying every bit of it.”*
	A15 Anonymity	Fans can anonymously participate in interactions and emotional expressions on digital media platforms.	*“Commenting without revealing my identity makes me feel more at ease—I can say whatever I want.”*

#### 3.3.3. Selective coding.

Selective coding is mainly based on the main categories to clarify the core categories and their typical relationship structure, and to form a complete and systematic explanatory framework, which often utilizes methods such as classification and abstraction [72]. In this study, the researchers conducted a comparative analysis and abstraction of the 15 initial categories and 6 main categories, and, combined with a retrospective analysis of the original materials, further identified 8 key pathways of influence, providing an empirical foundation for subsequent theoretical construction.

#### 3.3.4. Rigor.

To enhance the methodological rigor of this study, we adopted Lincoln and Guba’s [73] four criteria for evaluating qualitative research: credibility, transferability, dependability, and confirmability.

Credibility: First, a theoretical saturation test was conducted. Theoretical saturation refers to the point at which no new concepts, properties, or relationships emerge from the data [62]. An additional 886 comment entries were collected and subjected to three levels of coding, during which no new concepts or categories were identified, indicating that saturation had been reached. Second, the researcher engaged in long-term online observation of “sport fandom circle” and maintained reflective memos, which deepened contextual understanding. Third, data were drawn from multiple platforms—Weibo, Bilibili, and Xiaohongshu—allowing for contextual triangulation. Finally, several experts in the field of sports culture and communication were invited to conduct peer reviews of the coding results. No biases or inconsistencies in grounded coding were identified, further enhancing the reliability of the findings.

Transferability: This study provides a detailed account of sampling criteria, platform characteristics, and fan group profiles. In the case analysis, thick description of representative discourse is used to reconstruct the authentic context of fan emotional expression. These efforts aim to support future research in assessing the applicability of findings to other sports domains or sociocultural contexts.

Dependability: The entire process of data management, coding, and theory development was systematically documented using NVivo 11. A three-member research team—with backgrounds in communication studies, sociology, and fan culture [including one associate professor, one postdoctoral researcher, and one Ph.D. candidate, all formally trained in qualitative research]—jointly conducted the coding. The team first carried out joint preliminary coding on 10% of the sample to establish unified standards, followed by independent cross-coding, achieving an initial Cohen’s Kappa coefficient of 0.90. After the coding framework was established, the remaining 90% of the data were evenly divided, with each researcher independently coding 30%. To ensure consistency, multiple rounds of sample validation [using non-overlapping 10% subsets] were conducted in the mid and late stages of coding, yielding Cohen’s Kappa values ranging from 0.90 to 0.92, indicating high stability and inter-coder agreement. All data were coded in the original Chinese language to preserve the cultural and emotional nuances embedded in fan discourse. The initial translation of the research findings was conducted by bilingual researchers within the team, followed by independent proofreading and cross-validation by two native English speakers. For key terms used in the study, we adopted a back-translation approach to ensure the accuracy and consistency of terminology.

Confirmability: An independent memo system was maintained throughout the analysis to document researcher reflexivity, analytical reasoning, and decision-making processes. Theoretical development was supported with extensive original excerpts to minimize potential researcher bias and enhance transparency.

## 4. Results

**Fig 2:**
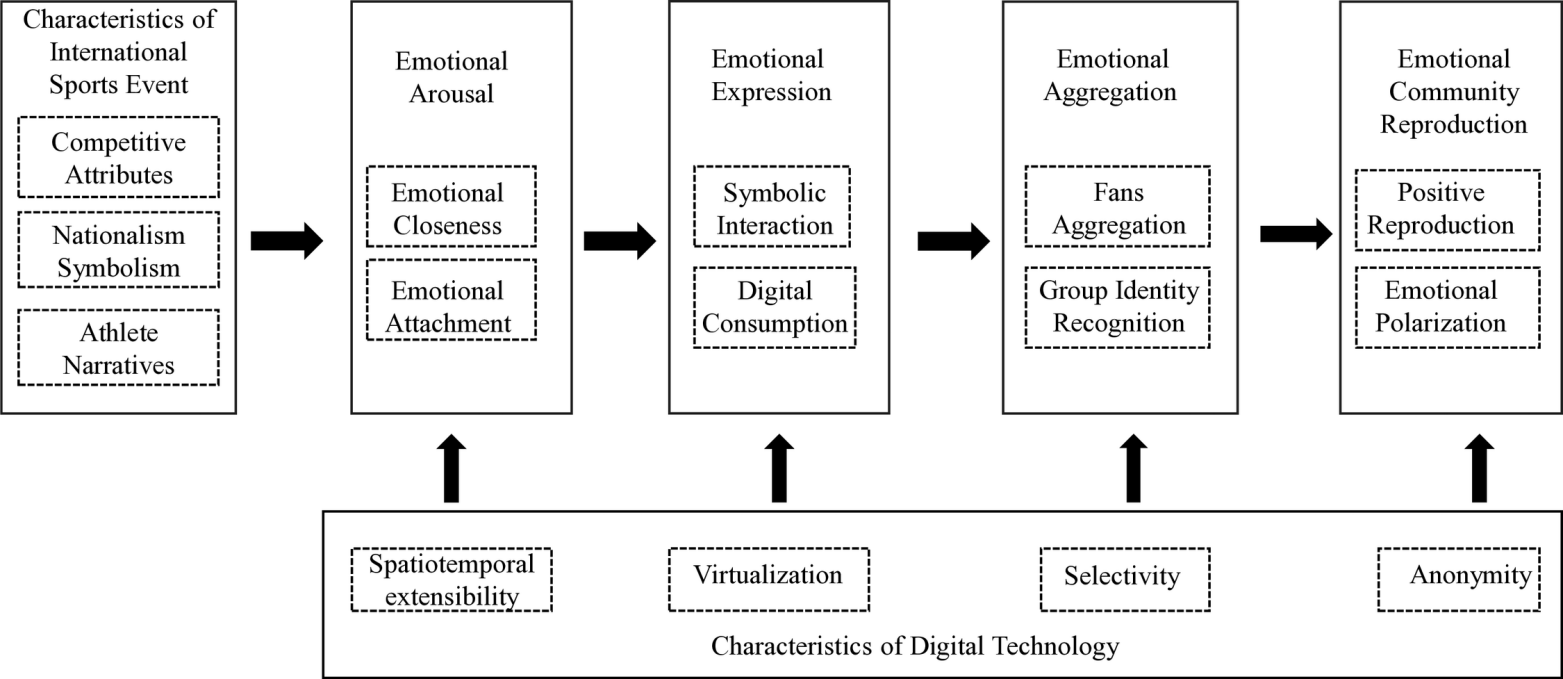
Model diagram of “sports fandom circle” formation and reproduction.

This study, through three levels of coding of the original data, distilled the storyline of “sports fandom circle” as emotional communities in the digital media era: their formation and reproduction are influenced by two key factors—triggering factors [Characteristics of International sports event] and enabling factors [Characteristics of digital technology]. The dynamic development of fan emotions follows four stages: emotional arousal, emotional expression, emotional aggregation, and emotional community reproduction. Specifically, the competitiveness of events, the symbolism of nationalism, and the narratives of athletes arouse fans’ emotions, shortening the emotional distance with athletes and creating emotional attachment. Fans express their emotions through symbolic interaction and digital consumption and, based on group identity, achieve emotional aggregation, forming highly cohesive emotional communities that ultimately enter the stage of emotional reproduction [including both positive reproduction and emotional polarization]. The spatiotemporal extensibility, virtualization, selectivity, and anonymity of digital technology empower this process, enabling fan emotions to transcend time and space constraints and achieve convenient interaction and expression. Based on this, this paper constructs the “structural model of sports fan circle formation and reproduction” [see Fig 2]. The following section of this paper will analyze the dynamic development mechanism of the emotional community of sports fan circles in detail, based on this model, combined with typical coding categories and fan discourse.

### 4.1. Key influencing factors in the emotional development of “sports fandom circle”

#### 4.1.1. Triggering factors: Characteristics of international sports event.

Social context plays a crucial role in triggering specific emotions—a process Turner refers to as “emotional arousal,” where individuals generate subjective responses based on non-realistic perceptions or expectations in particular situations [74]. This study reveals that emotional arousal in “sports fandom circle” stems from three distinctive characteristics of international sporting events: competitive attributes, nationalist symbolism, and athlete narratives, eventually leading to emotional focus on a particular star athlete. The Paris Olympics served as a paradigmatic case of this mechanism.

Competitive attributes, as the core characteristic of international sports events, is manifested in athletes’ behavior as they pursue victory in fair competition. In this study, both internal and external factors jointly stimulate the emotional arousal of fans. Among internal factors, the expectation of victory is a major driver of fans heightened emotional engagement, especially when the athlete they support reaches a critical match point. As one fan said: “*In the men’s singles quarterfinal of table tennis between Fan Zhendong and the Japanese player Tomokazu Harimoto, I really didn’t dare to blink for a second, just hoping he would win! It felt like I was standing right next to him, fighting together, and I completely forgot that I was just watching the game*.” [C2731] Among the external factors, the uncertainty of competitions provokes emotional fluctuations, transforming fans from spectators into “fellow fighters.” For example, one fan wrote: “*The match reversed several times, and each time I thought he was going to lose, but then it turned around again. My heart went up and down with him—it didn’t feel like I was watching someone else play; it felt like I was hanging in there with him until the very end*.” [C4027] At this point, the match result amplifies emotional fluctuations: victory brings both an enhancement of the athlete’s idol status and a collective carnival in the ‘fandom circle,’ while defeat can lead to emotional defense behaviors such as comment moderation and online arguments. For example, after Wang Chuqin’s loss in the men’s singles table tennis match at the Paris Olympics was questioned, his fans furiously retorted on Weibo: “*If it weren’t for the racket change, he would have won! If you don’t understand, just shut up*.” [C8710] Soon after, the Weibo comment section erupted in fierce fan battles between supporters and doubters.

Nationalism symbolism refers to the strong nationalistic sentiments embedded in international sports events, which are manifested through dimensions such as national symbols, linguistic and cultural expression, athlete identity, and political symbols. At the level of national symbols, items such as the national flag, national anthem, and national team uniforms are widely regarded as symbols of national honor and can strongly evoke emotional resonance among spectators at key moments. As one fan commented: *“When she stood on the podium in the red national team uniform, with the national anthem playing and the flag being raised, I truly felt that moment was so solemn that I was overwhelmed with tears.”* [C5231] Athletes, as representatives of the national image, often have their competitive achievements equated with national honor, and the audience’s emotions are subtly drawn closer to the competition arena. One fan said: *“He is not fighting for himself but for the entire country. In my heart, he is a national hero figure, the pride of our generation. At that moment, I really wanted to rush onto the podium and hug him.”* [C7456] At the level of linguistic and cultural representation, athletes’ use of their native language to express emotions and beliefs can effectively stimulate cultural identity and a sense of belonging among the audience. As another fan wrote: *“The first thing she said after the match was in Chinese, ‘Thank you, motherland.’ At that moment, I was really touched. Even though the match was held overseas, hearing my own language felt like hearing the voice of home.”* [C3884] At the level of political nationalism symbols, the confrontational structure between countries, the political significance carried by hosting rights, and the historical grievances that stir national emotions make some competitions imbued with meanings beyond sports itself. One fan expressed: *“This victory was so satisfying, especially against our long-standing rival. It felt not just like a sports competition, but like a battle for national dignity.”* [C6677] The study also found that international sports events attract a large number of non-sports fans through their nationalistic attributes. These fans are often supporters of a particular athlete and may not understand the sports themselves, focusing more on the athlete than the sport. For example, a non-sports fan who supports Fan Zhendong said in an interview: *“I don’t understand sports, but I like Fan Zhendong. His spirit of winning glory for the country on the international stage is something I will always follow and support.”* [C8129]

Athlete narrative refers to an emotional mode of expression centered around the athlete’s personal image, specifically encompassing their personal experiences, sportsmanship, and role model power. Athletes’ arduous journeys frequently evoke sympathy and recognition, fostering a deep emotional connection. For example, one fan commented: *“When he was young, his family was in a tough situation, and he started practicing table tennis before dawn every day. That kind of fighting spirit built up little by little really makes me feel sorry for him, and I understand better why he fights so hard.”* [C5021] Athletes’ perseverance and fighting spirit in critical moments evoke live emotional resonance from fans, bridging the emotional gap between them. One comment noted: *“She kept playing the entire match despite her injury. At that moment, I couldn’t hold back my tears—not because of how good she was, but because she was right there in front of us, gritting her teeth and fighting for the country and all of us.”* [C7543] In addition, the role model qualities displayed by athletes often inspire fans’ identification and psychological attachment, shifting their emotions from admiration to closeness. As one fan stated: *“Not only does she play well, but what’s even more valuable is that she always reminds everyone to respect their opponents and stay humble. Every time I hear her speak, I feel especially at ease, like someone is silently telling me how to be a better person.”* [C8629]

It is worth noting that this study found that, in order to attract traffic, many current media outlets excessively “heroize and mythologize” athletes during dissemination. For example, they portray athletes as “prodigy teenagers” or “invincible warriors” in a fixed narrative template, repeatedly emphasizing their “extraordinary talent” while neglecting the real process of systematic training and team collaboration behind them. Titles of such reports—including “She is destined to be a champion” and “Fan Zhendong is destined to be a legend”—frequently appear on short video platforms, trending Weibo topics, and marketing accounts’ posts. This type of narrative has altered fans’ emotional structures and focal points, shifting sports fans’ attention from performance on the field to the idealized worship of individual athletes. One fan of Sun Yingsha—who is also a table tennis fan and has participated in municipal-level table tennis competitions—frankly stated: *“After the Hangzhou Asian Games, I often came across narrative videos about Shasha and suddenly felt she was extraordinarily gifted. Now I’m her number one fan; I can’t stand anyone questioning her and will definitely fight back. Watching her matches feels like fulfilling my unfinished table tennis dream.”* [C9154]

#### 4.1.2. Enabling factors: Characteristics of digital technology.

Digital technology provides strong support for the development of the “sports fandom circle” and is a key enabling factor driving the dynamic development of fan emotions. This study identifies four main enabling mechanisms: spatiotemporal extensibility, virtualization, selectivity, and anonymity.

Spatiotemporal extensibility refers to how digital media transcends the limitations of time and space in communication, allowing fans to participate more conveniently in event interactions, thereby enhancing emotional arousal. In terms of temporal extension, fans can follow athletes’ updates, rewatch highlights, and participate in discussions of past games at any time, prolonging their emotional involvement. As one fan commented: *“I check her updates every day, and even after the match, I keep watching the highlights—I just grow fonder of her each time.”* [C5234] In terms of spatial extension, online platforms remove physical constraints, enabling users to interact in real time even when not at the venue. Through live information streams and collective feedback, they share a synchronized emotional experience. As one fan put it: *“Even though I’m just watching the livestream alone at home, seeing everyone’s comments fly across the screen and everyone responding together, it still feels like we’re watching the match together.”* [C8512]

Virtualization refers to the shift of sports content and interaction to online platforms, providing fans with a more convenient way to express emotions. It mainly includes content virtualization and dissemination virtualization. Content virtualization transforms match footage and athlete profiles into videos, images, and other formats, allowing fans to watch and save them anytime. As one fan said, *“It’s really convenient—match videos and photo collections are just a quick search away.”* [C5956] Dissemination virtualization relies on social media platforms to enable real-time interaction, where fans can express their emotions instantly through bullet comments and replies. As one fan shared, *“The moment she scored, I sent a bullet comment right away—it felt like everyone was excited together.”* [C6052]

Selectivity refers to the ability of users in the digital media environment to actively choose content that interests them, while platforms also offer personalized recommendations based on user behavior through algorithms. Fans tend to focus on specific figures or topics, fostering emotional convergence within the group. As one fan commented: “*I joined several groups of people who like Fan Zhendong — we share his videos and interviews every day, and it really feels like being part of a community.”* [C8968] Algorithmic recommendations analyze users’ browsing and interaction records to automatically push related content, which may lead to the phenomenon of an “information cocoon.” In such cases, fans’ attention becomes confined to a particular athlete, and their emotional tendencies are continually reinforced. For example, one fan noted: *“After I clicked on one of Fan Zhendong’s match videos, my homepage was filled with his highlights and interviews for days. The platform knows me so well, but I’m enjoying it.”* [C7391]

Anonymity refers to the ability of digital media platforms to allow users to conceal their real identities, which provides fans with a sense of psychological security, making them more willing to vent emotions and express their views. As one fan mentioned: *“When I see someone belittling my favorite player, I’ll use another account to argue back—after all, no one knows it’s me.”* [C1342] In addition, an anonymous environment also facilitates the rapid spread of extreme emotions, intensifying online confrontations. One fan admitted: *“As soon as it hit the trending topics, it was all anonymous accounts arguing. After seeing it so much, I ended up replying a few times too.”* [C3621]

### 4.2. The dynamic emotional development path of fans in “sports fandom circle”

Under the influence of the aforementioned external factors, fans’ emotions in “sports fandom circle” exhibit a four-stage development path: “emotional arousal—emotional expression—emotional aggregation—emotional community reproduction.”

#### 4.2.1. Emotional arousal.

Emotional arousal marks the initial stage in the formation of a fan emotional community. The characteristics of international sports events trigger this emotional response, while the spatiotemporal extensibility of digital technology breaks traditional constraints, allowing fans to project their emotions onto idols anytime and anywhere, thereby shortening the emotional distance between them and the athletes, mainly manifested in the establishment of a sense of closeness, resonance, and participation. The sense of closeness arises from increased contact frequency, as fans gradually develop a sense of familiarity through likes, comments, and live interactions—leading to feelings such as *“he feels like a friend to me”* [C3321] Resonance stems from emotional responses to athletes’ struggles and efforts, as one fan noted: *“Seeing him giving his all for his dreams, I really understand.”* [C4598] Participation refers to fans’ sense of being part of the athlete’s journey through long-term support and “accompanying” the athlete, with continuous attention to a particular athlete leading to a stronger sense of participation. Some fans even develop “relationship fantasies. These fantasies typically take two forms: one is the “friend-like companionship,” where fans see the athlete as a close friend growing alongside them, as a fan shared: *“Witnessing Ma Long grow from an inexperienced player to a mature athlete feels like accompanying him along the way.”* [C1124] The second is the “romantic fantasy,” where fans project their ideal emotions onto the athlete, often referring to them as “husband” or “wife”: *“My heart races every time he comes out to play.”* [C2783] According to a survey by the People’s Forum Questionnaire Center [N = 3895], 18% of respondents admitted to having some degree of “relationship fantasy” toward their idols [75]. This phenomenon indicates that digital platforms have reshaped the emotional distance between fans and athletes.

As the emotional distance narrows, fans’ emotions further transform into emotional attachment, including achievement attachment and personal attachment. Achievement attachment refers to the emotional connection fans develop through continuous attention and admiration for athletes’ outstanding athletic abilities. *“The way he controls the game during matches is so captivating; every move is precise and powerful—I can’t get enough of it! His technical skills really amaze me, and I get completely absorbed. If I don’t watch his match videos every day, I feel empty inside, like something’s missing.”* [C2714] In contrast, personal attachment refers to the emotional connection fans develop through deep identification with athletes’ personal qualities and spiritual values, treating them as an emotional anchor. *“No matter whether he wins or loses, just seeing him smile on the court makes everything worthwhile. His resilience and gentleness have touched me, and whenever I face difficulties in life, thinking of him gives me strength. I feel like he has long been a part of my life.”* [C3329] This study also found that fans’ attachment can shift from achievement attachment to personal attachment. As one fan expressed, *“After watching Fan Zhendong’s match against Tomokazu Harimoto, I was really shocked by his skills, and since then I’ve followed every one of his matches. But the more I follow him, the more I discover that he’s not only an amazing player, but also a genuinely sincere and warm person off the court. Now I like him not just for his skills, but also for his perseverance and charismatic personality. Every time there’s an offline event, I always go to support and check in.”* [C4782] This phenomenon is similar to that found in entertainment fandoms, where fans’ obsession with idols goes beyond appreciation of their work and stems from infatuation with their persona [76].

#### 4.2.2. Emotional expression.

After experiencing emotional arousal, the emotions internally triggered in fans do not remain at the level of feeling alone but are instead expressed through concrete actions. In classic fan subculture research, fans’ emotional expression is characterized by “productive consumption,” where they not only keep up with the latest developments of the original work but also actively engage in secondary creations [77]. However, this form of emotional expression is mainly confined to subcultural groups and realized through specific physical media, which is not conducive to organizational mobilization. The arrival of the digital age has virtualized the content and form of dissemination, fundamentally breaking this limitation. Fans’ emotional expression is no longer restricted to physical products [such as live viewing] but is extended through symbolic interaction and digital consumption.

Symbolic interaction refers to the way individuals convey emotions and express identity through recognizable symbolic systems, typically including three types: visual symbols, linguistic symbols, and behavioral symbols. During the Paris Olympics, fans vividly expressed their emotions using visual symbols such as emojis, GIFs, and event posters: *“Every time Fan Zhendong wins a point, I take a screenshot and make a GIF of that moment when he swings his racket. Everyone says it’s so exciting—it feels more powerful than just posting words.”* [C1845] Through linguistic symbols such as nicknames, slogans, and catchphrases, fans enhanced interactive fun: *“We all call Sun Yingsha ‘Little Tank Shasha’—when she charges, she’s so unstoppable. Just shouting that nickname can instantly fire everyone up, and the comment section goes wild.”* [C2912] Fans also participate in emotional expression and collective rituals using behavioral symbols like reposting, liking, and voting to support athletes: *“Before Wang Chuqin’s match, I always repost his official account’s posters and get my friends to like it. If I don’t repost or like it, I feel like I’m not really part of today’s match.”* [C3790]

Digital consumption is a key external form of fan emotional expression, mainly including content consumption, product consumption, and support-based consumption. The first two typically refer to spending related to sports events and merchandise. As one fan shared: *“I bought that limited edition NFT badge of his and the official 3D signature card. Even if I can’t use them, just collecting them makes me happy.”* [C2194] This study finds that support-based consumption is a defining feature that distinguishes the “sports fandom circle” from ordinary sports fans. The celebrity fandom model from the entertainment industry has extended into the “sports fandom circle”, where fan spending has shifted from simply buying merchandise to investing in areas that align with the athlete’s development needs. This helps shape the athlete’s ideal persona while giving fans emotional satisfaction. As one fan put it: *“It’s not that I really need these things; it’s that he needs popularity and exposure, and I’m willing to spend money to help him top the charts.”* [C3978] This consumption model has gradually transformed the traditional fan “gift economy” into a “digital donation economy.” As a result, in the “sports fandom circle”, fans supporting athletes through “cheering activities” has become increasingly common, such as purchasing digital tokens like “little red flowers” or “adding energy” on popularity charts. One fan wrote: *“I’ve already given 50 little red flowers today; I hope he can reach number one.”* [C4601] It is evident that fans now regard their own “spending power” as a key indicator of the athlete’s popularity and commercial value, and that the act of consumption itself has become a symbol of their emotional identity.

#### 4.2.3. Emotional aggregation.

Building on the above two levels, the construction of emotional communities in “sports fandom circle” also requires emotional aggregation among fans. Digital technology, through mechanisms of active selection and algorithmic recommendation, accelerates emotional aggregation by prompting fans to cluster and form group identity in virtual environments.

The foundation of emotional aggregation lies in fan gathering. Traditional sports fans are limited by media constraints, with single forms of interaction and difficulty crossing community boundaries. Fan gathering mainly takes place offline. Even today, in the “sports fandom circle”, some fans with strong personal attachment still prefer offline gathering to express their emotional support. As one fan shared: *“I traveled from the north to the south just to watch Dong Ge’s match. Half the stadium was filled with fans in matching outfits, cheering in unison—the atmosphere was electric. I also met like-minded friends there, and the whole trip was truly worth it.”* [C3021] What’s different is that in the digital age, the formation and expansion of “sports fandom circle” is faster, and communication between fan groups is more convenient. It’s easier to form online communities, where each community generally comprises fans of the same athlete, so there are many shared topics, making it easy to resonate with each other. Frequent interactions in these communities enhance the sense of belonging and accelerate emotional aggregation. As one fan commented: *“At first, I just liked Dong Ge by myself, but after joining the fan group, I realized there were so many like-minded people. Every day, everyone passionately discusses his matches and highlights—even just one photo can be talked about for a long time. The atmosphere is so contagious. Before I knew it, I started editing videos and writing posts to participate. Although many people have never met in person, the feeling of chasing our idol together is like fighting side by side. In this group, I truly found a sense of belonging—it’s like a big, united family.”* [C3920]

The core of emotional aggregation lies in the establishment of group identity. Through interactions based on shared interests and emotions, fan communities continuously deepen their cultural identity, social identity, and value identity related to their idols and the fandom circle they belong to, thereby constructing a stable and exclusive emotional community. Cultural identification refers to fans’ acceptance of fandom rules and atmosphere, as well as the formation of a shared sense of identity and belonging across diverse backgrounds. As one fan said: *“No matter if we come from the south or the north, we are all guardians of Fan Zhendong. We cannot bring negativity to him; everyone in the group consciously abides by the rules.”* [C5832] Social identification is reflected in fans building emotional connections with others through their shared support for an idol, gaining understanding and acceptance, which helps them escape feelings of isolation and reinforces their sense of belonging. As one fan put it: *“When I used to follow idols alone, I always felt lonely and misunderstood. But after joining the Sun Yingsha fan group, chasing matches, voting, and chatting together made me feel the warmth of being understood, and it made me identify more strongly as a ‘Shafan.’ It made me more determined to stay in this circle.”* [C6417] Value identity stems from fans’ recognition of both the athlete and the values of the fandom circle, gradually transforming into emotional cohesion within the group and reinforcing fans’ positions as “exclusive fans.” As one fan commented: *“Seeing Dong Ge participate in an educational support project really touched me. Not only is he a great player, but he’s also a responsible person. At that moment, I felt even more determined to be his fan only, and I felt proud of the atmosphere in our circle.”* [C6923]

This study also found that once group identity is formed, fan communities often develop a set of standardized strategies to support athletes through identity symbols and organizational models. First, they create identity tags [such as Fan Zhendong fans calling themselves “Fanxing”] to strengthen their sense of identity and facilitate the organization of support activities. Second, collective actions are often implemented in a highly organized manner, leveraging digital technology to build a rationalized bureaucratic system characterized by clear divisions of labor and hierarchical structures. This structure typically takes the form of concentric circles, where core “big fans” are responsible for decision-making and resource coordination, the next core layer of fans handles content production and promotion, and the peripheral fans drive specific activities. Particularly during large-scale events such as the Olympics, fan organizations spontaneously form collaborative models to ensure that support activities proceed efficiently and in an orderly manner.

#### 4.2.4. Emotional community reproduction.

As the emotional communities of sports fans continue their self-reproduction, their influence on the public sphere grows increasingly significant. This study finds that whether the reproduction of “sports fandom circle” reinforces the power of sports or disrupts its order depends on how fans utilize the anonymity of digital technology. Some fans use anonymous platforms to enhance the quality of discourse and foster a constructive collective consciousness, channeling emotions toward rational expression and collective interests. Others, however, exploit anonymity to escape real-world constraints, leading to extreme emotional expression, undermining rational interaction, and even triggering conflict that challenges public order.

(1) Positive Reproduction of Fan Emotions

In a highly individualized society, the “sports fandom circle” provides fans with opportunities to reintegrate into a collective. Through digital platforms, fans can experience and understand different life states and gradually acquire the ability to reflect on their own emotional practices [78]. The anonymity and openness of these platforms create new spaces for fan interaction and promote emotional expressions that align with social expectations, particularly in terms of sports fairness and cultural dissemination.

In some public issues, “sports fandom circle” also demonstrate rational voices, especially in constructing fairness in participation rights and discourse power. Regarding gender equity in competition, fans have used platforms like Weibo Super Topics to advocate for the rights of female athletes. During the controversy over transgender participation in the Paris Olympics, some fans called for “more professional participation standards to ensure fairness for female athletes.” Meanwhile, female fans compiled similar cases and educational materials, emphasizing: *“We support the rights of transgender individuals, but women’s events must ensure fairness for female athletes.”* [C5247] In terms of regional equality, fans have engaged in cheerleading and comment management for athletes from underprivileged areas, guiding public discourse to draw attention to marginalized groups. One fan wrote in a support post: *“Athletes from poor regions have just as much talent, and we should fight for more visibility for them.”* [C6174] Regarding sports discourse power, female fans spoke up for women athletes: *“Every time she swings her paddle, she’s telling us that girls can also dominate the game. Our voting and moderation are not just for her achievements but also to fight for every hardworking girl.”* [C6938]

In addition, fans have contributed to the spread and recognition of sports culture by sharing athletes’ stories and organizing group viewing events. One fan remarked, *“Seeing these athletes work so hard for their dreams, we should give them a thumbs up and learn from their sportsmanship.”* [C5236] Such collective efforts have also brought attention to lesser-known sports. *“I never watched archery before, but my friend dragged me to watch a match, and I was immediately hooked by the intense atmosphere.”* [C5889] *“We’re not professional promoters, but we’re willing to use our passion to let more people see the value of these lesser-known sports.”* [C6720]

(2) Polarization of Fan Emotions

The phenomenon of emotional polarization in the “sports fandom circle” shows a complex structure, mainly reflected in the opposition between fan groups, emotional conflicts between fans and athletes, and tensions between fans and commercial brands.

The opposition between fan groups is the most direct manifestation of emotional polarization within the “sports fandom circle”, especially during high-pressure international competitions. The anonymity of digital spaces removes real-world identity constraints, amplifies emotional expression, and weakens rational discussion, ultimately leading to opposing camps [79]. Some fans attack each other due to differing stances—for instance, one comment reads: *“Wang Chuqin’s fans are once again excusing his mistakes after his loss—at the end of the day, he’s just not as steady as our Dong Ge.”* [C187] In athlete Super Topics, long-standing debates such as “Who’s the real No.1” and “Who dragged the team down” have sparked internal disputes. As one fan lamented: *“I came to watch the match, but every day feels like a battlefield.”* [C624] Another fan remarked: *“Seeing them trashing my favorite player again, I just switched accounts and clapped back. I couldn’t be bothered to reason with them anymore.”* [C932]

Emotional conflicts between fans and athletes are another manifestation of polarization, primarily stemming from the gap between fans’ high expectations and athletes’ actual performance. Anonymous platforms fuel emotional outbursts, with some fans turning to cyberbullying out of disappointment—particularly among those with strong achievement-based attachment to the athlete. For example, after Wang Chuqin was eliminated in the round of 32 in the men’s singles at the Paris Olympics and continued to lose in subsequent events, he faced harsh criticism such as “wasting national resources” and “setting new lows.” One fan remarked, *“I used to watch every one of his matches and even defended him against haters, but after his Olympic exit and continuous losses, I’m just completely disappointed.”* [C1580]

The tension between fans and commercial brands represents the third dimension of emotional polarization within the “sports fandom circle”. As sports events become increasingly intertwined with brand partnerships, fan emotions are also tied to commercial interests. Brands leverage emotional marketing to engage fans, while fans express support through endorsements and consumption. However, when brand actions fall short of fan expectations, emotional support can quickly turn into emotional conflict. Take Li-Ning as an example: the long-established “national brand” image it cultivated backfired during the Paris Olympics. Design flaws in its sponsored national team uniforms—such as poor sweat absorption and discomfort—sparked collective backlash from fans. One fan of Fan Zhendong complained, *“The clothes stuck to his body and affected his performance—Li-Ning has betrayed the athletes’ trust.”* [C2381] The dissatisfaction escalated into a large-scale boycott of the brand. Additionally, athletes’ commercial endorsements can also trigger fan discontent. Some questioned, *“Constant ad shoots are bound to affect training quality,”* [C3062] blaming a decline in performance on excessive commercial engagements. This protective anxiety often translates into criticism of the sponsoring brands.

## 5. Discussion

Digital media has transformed sports communication, giving rise to emotionally driven fan communities centered around sports celebrities—known as the “sports fandom circle.” Previous studies largely emphasize fan belonging [23,24], loyalty [26,27], and consumption practices [29] in group formation. However, there is a lack of systematic research on the dynamic development of emotions. In terms of case selection, prior research has mainly focused on traditional fan groups motivated by athletic performance, exploring their online interaction patterns [48], behavioral typologies [27], and extreme actions [80]. In contrast, this study examines fan communities that are intensely devoted to athletes and exhibit high levels of organization and social stratification. This shift in perspective not only changes the research subject but also broadens the analytical scope, offering a deeper understanding of emotionally driven group behavior.

This study adopts the grounded theory method, using the 2024 Paris Olympics as the research context, and centers on the core question: “In the context of digital media, how do “sports fandom circle” form and reproduce from the perspective of fan emotional development?” The study proposes a theoretical model of the dynamic development of fan emotions, which carefully delineates the key influencing factors and dynamic development path of fan emotions. The main research findings will be further discussed through comparison with existing related studies.

### 5.1. What are the key factors influencing the emotional development of fans within the “sports fandom circle” in the digital era?

This study identifies two key categories of influencing factors: the characteristics of international sports events [triggering factors] and the characteristics of digital technology [enabling factors]. Regarding the triggering factors, previous research has confirmed that elements such as the competitiveness of international sports events [81,82], nationalistic symbolism [83–85], and athlete-centered narratives [86,87] can all evoke emotional arousal among audiences. The findings of this study are consistent with this view, as these three factors likewise trigger emotional arousal among fans within the “sports fandom circle”. However, unlike the aforementioned studies that mainly focus on general audiences or ordinary sports fans, this study further reveals the uniqueness of emotional arousal within the “sports fandom circle”: First, the “individual projection” of nationalistic sentiment—fans in the “sports fandom circle” are more inclined to project their national emotions onto individual athletes rather than onto teams or tournaments, as is more common among traditional sports fans. This dynamic has even attracted a large number of non-sports fans to emotionally engage and exhibit obsessive behaviors. One possible explanation is that, compared to sports fans, non-sports fans—lacking professional knowledge—find it difficult to resonate with teams or competitions on a deeper level, while the personal traits of athletes offer more intuitive appeal and thus become more natural carriers of emotional projection [88]. Second, the emotional reconstruction effect of media narratives about athletes—while existing studies acknowledge the emotional value of athlete storytelling [89–91], they have not sufficiently explored how the narrative style reshapes fans’ idealized perceptions of athletes. This study points out that the media’s use of “heroic” and “deified” storytelling about athletes is reshaping fans’ emotional structures, shifting their focus from the competitive aspects of sports to an excessive idealization of individuals. A possible underlying reason for this phenomenon is that such narratives satisfy audiences’ needs for emotional resonance and identity affirmation [92].

As for the enabling factors, this study identifies four key technological mechanisms—spatiotemporal extension, virtualization, selectivity, and anonymity—which together foster the sustained development of fan emotions. This aligns with the broader judgment of social platforms’ “emotional amplification effect” [93], yet prior research has lacked a systematic analysis of the micro-level technological mechanisms behind this effect. In contrast, this study further refines the understanding of how these mechanisms function and constructs a staged and structured analytical pathway. The relevant analysis will be integrated with the emotional development trajectory proposed by the model.

### 5.2. What is the dynamic path of emotional development of fans within the “sports fandom circle” in the digital era?

This study proposes a four-stage model—emotional arousal, emotional expression, emotional convergence, and emotional community reproduction—to illustrate the dynamic process of emotional development among fans in the “sports fandom circle”. The specific stages are analyzed as follows:

In the emotional arousal stage, the combined effect of international sporting events and digital technologies bridges the emotional distance between fans and athletes, fostering attachment—a finding consistent with traditional conclusions about “mega sporting events triggering emotional arousal” [94]. This study further reveals that the spatiotemporal extensibility of digital technologies [e.g., live streaming, short video interactions] redefines engagement boundaries, enabling rapid virtual empathy-based attachment formation [95,96]. The emotional attachment of fans in the “sports fandom circle” follows a distinct “achievement attachment → personal attachment” trajectory. While aligning with Harasta’s [97] “identification-to-loyalty” progression, in which fans begin with individual identification, then build emotional connections through social media, ultimately becoming highly loyal “hardcore fans.” This trajectory underscores that emotional attachment in “sports fandom circle” is a psychological mechanism that gradually deepens through media interaction, corroborating Valero’s [98] concept of projection from the “objectified other” to the “extended self.” In this process, fans’ attachment to athletes shifts from viewing them as carriers of achievement to projecting onto them an idealized self, thereby blurring the boundaries between fan and athlete.

In the emotional expression phase, fans externalize their inner emotions through symbolic interactions and digital consumption. This aligns with existing research on fan-idol relationships in the internet era regarding fan cultural participation [99]. However, while prior studies categorized fan expressions as either cultural engagement or aesthetic practice [42,100], our study reveals that the symbolic interactions in “sports fandom circle” transcend mere content participation—they serve as vehicles for fans to assert personal stances, gain social recognition, and construct group identities [101]. In traditional sports fan studies, digital consumption is typically seen as rooted in preference and identity, such as purchasing merchandise to show support [26,28,29]. However, this study reveals that digital consumption in the “sports fandom circle” has evolved beyond traditional purchasing behavior, transforming into a form of “identity-symbolic capital operation.” Practices like “voting campaigns” and “fan support initiatives” monetize emotional intensity [46], a phenomenon adapted from entertainment fandom [e.g., K-pop chart manipulation culture [102]], to reinforce their sense of subcultural belonging. In “sports fandom circle”, this capital-driven expression fosters a new “community status competition mechanism” where emotional investment becomes the core metric of social hierarchy.

In the emotional aggregation phase, digitally constructed “information cocoons” channel fans’ attention toward specific athletes’ content [103], intensifying emotional resonance and accelerating community formation and group identity. This aligns with Brady et al.’s [104] findings on algorithmic recommendations facilitating information convergence. Our study further reveals that collective identity in “sports fandom circle” is co-constructed through a tripartite framework of cultural, social, and value-based identification, highlighting the social embeddedness and value-oriented nature of fan identity. This challenges prior conceptualizations that reduced sports fan allegiance to singular attachments to teams or match outcomes [23,105]. Notably, this study also finds that “sports fandom circle” show a rational hierarchical structure, with “big fans” coordinating resources and peripheral members executing tasks. This organizational pattern aligns with the concentric-circle logic typical of entertainment fandoms [13], it starkly contrasts with the decentralized, loosely-knit nature of traditional sports fan communities [33].

In the reproduction of emotional community phase, this study finds that anonymous platforms foster fans’ moral courage on social media, promoting sports justice and cultural exchange. Such platforms show positive effects consistent with prior studies, including lowering expression barriers, encouraging public engagement, and enabling voices against injustice [106,107]. At the same time, de-identification may intensify emotional polarization and group antagonism, echoing concerns about diluted responsibility and moral deviance in anonymous settings [108,109]. This finding challenges traditional view that attribute community continuity primarily to structural stability or organizational inertia, such as the maintenance of groups through institutional arrangements and habitual practices [110].

In summary, the four-phase model of fan emotional development in “sports fandom circle” proposed by this study demonstrates clear sequential progression: emotional awakening establishes the perceptual foundation for fan engagement; emotional expression externalizes and extends these awakened feelings, granting individual emotions visibility and interactivity; emotional aggregation transforms dispersed expressions into collective identity, creating conditions for community formation; and ultimately, the reproduction of emotional communities sustains and perpetuates this affective structure. The model is consistent with existing research on the role of emotion in identity construction and collective action [49,111], it goes beyond the limitations of the traditional “static attribution–behavioral outcome” framework. In conventional models, emotion is often treated as an antecedent variable that facilitates identity formation or collective action, with a primary focus on one-time effects or immediate emotional responses—such as how a specific emotion influences identity recognition or action intention [26,31,32]. However, these studies overlook the dynamic evolution of fan emotions in the context of social media and its impact on group organizational structures. In contrast, this study addresses that gap by emphasizing the continuity and stages of emotional development, proposing a more process-oriented analytical framework.

The theoretical contributions of this study are threefold. Firstly, it proposes a dynamic developmental model of fan emotions, enriching the theoretical foundation of “sports fandom circle” research while providing a systematic framework for understanding emotion-driven participation logic in digital communities. Secondly, it refines the micro-mechanisms of emotional arousal in international sporting events, revealing the unique role of “individual projection of national sentiment” and “heroization narratives” within fandom contexts, thereby deepening our understanding of the heterogeneity in “sports fandom circle”. Lastly, the study moves beyond abstract discussions of digital media’s role by precisely delineating the operational mechanisms of technological platforms in emotion generation, expression, and community construction, thus expanding the interdisciplinary boundaries between affective communication and media studies.

Based on the above theoretical findings, practical implementation requires collaborative efforts among media institutions, digital platforms, sports institutions, and individual fans to guide the rational expression of fan emotions and foster healthy community interactions. First, sports narratives should be enriched to move beyond singular emotional mobilization. Media and event organizers should highlight athletes’ hard work and professionalism in their coverage, rather than framing “bringing glory to the nation” as the sole narrative focus. Athletes themselves should also actively share their training processes, setbacks, and personal reflections to break idealized projections such as the “flawless persona.” Second, platform governance should establish a multidimensional framework that includes algorithm optimization, content moderation, behavioral guidelines, and accountability mechanisms. Platforms can curb the over-dissemination of emotionally charged content by adjusting recommendation algorithms, adding comment prompts, enhancing human oversight, and setting up user reporting and tiered response systems. These measures also help strengthen users’ awareness of responsible expression. In addition, clear codes of conduct should be applied to influencers and high-profile creators, with tiered penalties for violations. To ensure transparency and accountability, platforms should regularly publish governance reports and be subject to public and third-party oversight.

## 6. Conclusions

In the digital media era, the “sports fandom circle” exemplifies profound transformations in fan engagement modalities. This study analyzes the developmental trajectory of fan emotions within the “sports fandom circle”, aiming to elucidate its formative mechanisms. Findings reveal that the characteristics of international sporting events and the characteristics of digital technologies play pivotal roles in shaping the development of fan emotions, thereby delineating a dynamic developmental process encompassing “awakening-expression-aggregation-reproduction.” Building upon these insights, this study proposes the “Dynamic Emotional Development Model of ‘Sports Fandom Circle’”. The model not only addresses current research gaps regarding the emotional progression in “sports fandom circle” but also provides theoretical foundations and empirical references for comprehending the generative logic and governance practices of fan culture on digital platforms, demonstrating significant academic value and practical relevance.

It is worth emphasizing that although this model is constructed based on the Chinese context, it possesses a certain degree of cross-cultural applicability. International sports events, as powerful emotional stimuli, are prevalent across cultures—for instance, European football fans’ passion for the World Cup [112] and NBA fans’ emotional resonance with star narratives serve [113] as compelling examples. Meanwhile, international mainstream social media platforms such as YouTube, TikTok, and Instagram share functional similarities with Chinese platforms, including live streaming, short video creation, and personalized recommendations. Studies conducted abroad have also shown that such platforms exert a significant influence on user emotions [93,104,114,115]. Although fans’ emotional practices may differ across cultural contexts, existing research has shown that global fan cultures exhibit certain commonalities in the logic of emotional development [116]. This provides a theoretical foundation for the cross-cultural applicability of the dynamic emotional development pathway proposed in this study.

Despite its cross-cultural applicability, the model also faces limitations stemming from cultural contexts in practical application. On one hand, Chinese fan culture is influenced by collectivist values, where emotional expression emphasizes group orientation and national identity [45,117,118]. In contrast, fans in individualistic or culturally pluralistic countries may place greater emphasis on personal emotions and local affiliations [45], which could lead to variations in the emotional aggregation pathway. On the other hand, China’s state-led sports system imbues sports events with strong national honor symbolism, making nationalism a primary emotional trigger [118,119]. In Western contexts, although national identity exists, emotional arousal is more often driven by club culture, local identity [120,121]. Therefore, the role of “nationalism” as an emotional activation mechanism in the model may be diminished in overseas settings. Accordingly, applying the model requires contextual adjustments to suit specific cultural environments.

In addition, this study has certain limitations in terms of research methodology and scope. First, while the use of qualitative analysis helps to gain an in-depth understanding of emotional mechanisms, it lacks quantitative validation. Future research could incorporate empirical analysis through social media data and affective computing methods to enhance the objectivity and generalizability of the findings. Second, the data sources are primarily drawn from Weibo, Xiaohongshu, and Bilibili. Although these platforms offer advantages such as diverse user bases, rich content types, and distinctive fan interaction features, the exclusion of other platforms like Douyin and Zhihu may limit the comprehensiveness of emotional expression characteristics. Future studies could expand the dataset accordingly for a more holistic analysis. Third, this study mainly focuses on fan behavior during major international sports events, with limited attention to everyday sports events and niche vertical communities. Future research could broaden the contextual scope to enhance the model’s broader applicability and practical relevance.

## Supporting information

S1 Table A list of 50 accounts (40 primary accounts and 10 supplementary accounts). (DOCX)
