# Supplementary material for: The formation mechanism of “sports fandom circle” in the digital media era: An analysis from the perspective of fan emotional dynamic development
Source: PLoS One. 2025 Sep 3;20(9):e0330900. doi: 10.1371/journal.pone.0330900 (PMC12407401; doi:10.1371/journal.pone.0330900)
Supplement: S1 Table — (DOCX) [file pone.0330900.s001.docx]

| Primary Accounts | | |
| --- | --- | --- |
| Serial Number | Follower Count | Number of Posts |
| User_1 | 10000-50000 | 500-1000 |
| User_2 | 10000-50000 | 100-500 |
| User_3 | 10000-50000 | 1000-5000 |
| User_4 | 10000-50000 | 100-500 |
| User_5 | 10000-50000 | 10-100 |
| User_6 | 10000-50000 | 100-500 |
| User_7 | >3000000 | 10-100 |
| User_8 | 10000-50000 | 1000-5000 |
| User_9 | >3000000 | 10-100 |
| User_10 | 10000-50000 | 100-500 |
| User_11 | 10000-50000 | 100-500 |
| User_12 | 10000-50000 | 10-100 |
| User_13 | 10000-50000 | 100-500 |
| User_14 | 10000-50000 | 100-500 |
| User_15 | 10000-50000 | 100-500 |
| User_16 | 10000-50000 | 100-500 |
| User_17 | >3000000 | 10-100 |
| User_18 | >1000000 | 1000-5000 |
| User_19 | 500000-1000000 | 1000-5000 |
| User_20 | 50000-250000 | 1000-5000 |
| User_21 | 50000-250000 | >5000 |
| User_22 | 10000-50000 | >5000 |
| User_23 | >3000000 | 100-500 |
| User_24 | >3000000 | 100-500 |
| User_25 | 50000-250000 | 1000-5000 |
| User_26 | 50000-250000 | >5000 |
| User_27 | 50000-250000 | 100-500 |
| User_28 | 50000-250000 | 1000-5000 |
| User_29 | 50000-250000 | 500-1000 |
| User_30 | 50000-250000 | 1000-5000 |
| User_31 | 10000-50000 | >5000 |
| User_32 | 10000-50000 | 1000-5000 |
| User_33 | 50000-250000 | >5000 |
| User_34 | 10000-50000 | 500-1000 |
| User_35 | 10000-50000 | 10-100 |
| User_36 | 10000-50000 | 100-500 |
| User_37 | 10000-50000 | 10-100 |
| User_38 | 10000-50000 | 10-100 |
| User_39 | 50000-250000 | >5000 |
| User_40 | 10000-50000 | 100-500 |
| Supplementary Accounts | | |
| Serial Number | Follower Count | Number of Posts |
| User_1 | 1000-3000 | 100-500 |
| User_2 | 1000-3000 | 100-500 |
| User_3 | 1000-3000 | 100-500 |
| User_4 | 3000-5000 | 100-500 |
| User_5 | 3000-5000 | >5000 |
| User_6 | 1000-3000 | 10-100 |
| User_7 | 1000-3000 | 10-100 |
| User_8 | 1000-3000 | 100-500 |
| User_9 | 1000-3000 | 100-500 |
| User_10 | 3000-5000 | 10-100 |
